# Design of Hollow Porous P-NiCo_2_O_4_@Co_3_O_4_ Nanoarray and Its Alkaline Aqueous Zinc-Ion Battery Performance

**DOI:** 10.3390/ijms242115548

**Published:** 2023-10-25

**Authors:** Zhe Liang, Chenmeng Lv, Luyao Wang, Xiran Li, Shiwen Cheng, Yuqiu Huo

**Affiliations:** Department of Chemistry, School of Science, Northeastern University, Shenyang 110819, China; liz202310@163.com (Z.L.); 2100281@stu.neu.edu.cn (C.L.); 2100285@stu.neu.edu.cn (L.W.); 2200296@stu.neu.edu.cn (X.L.); 2200286@stu.neu.edu.cn (S.C.)

**Keywords:** P-NiCo_2_O_4_@Co_3_O_4_, ion etching, alkaline aqueous, high-power energy, zinc-ion battery

## Abstract

Alkaline aqueous zinc-ion batteries possess a wider potential window than those in mildly acidic systems; they can achieve high energy density and are expected to become the next generation of energy storage devices. In this paper, a hollow porous P-NiCo_2_O_4_@Co_3_O_4_ nanoarray is obtained by ion etching and the calcination and phosphating of ZiF-67, which is directly grown on foam nickel substrate, as a precursor. It exhibits excellent performance as a cathode material for alkaline aqueous zinc-ion batteries. A high discharge specific capacity of 225.3 mAh g^−1^ is obtained at 1 A g^−1^ current density, and it remains 81.9% when the current density is increased to 10 A g^−1^. After one thousand cycles of charging and discharging at 3 A g^−1^ current density, the capacity retention rate is 88.8%. Even at an excellent power density of 25.5 kW kg^−1^, it maintains a high energy density of 304.5 Wh kg^−1^. It is a vital, promising high-power energy storage device for large-scale applications.

## 1. Introduction

The redox potential of zinc metal in an alkaline aqueous is −1.44 V; therefore, zinc-based batteries with alkaline electrolytes have a higher operating voltage and a higher energy density. They have a lower redox potential than mildly acidic electrolytes (−0.763V), which can expand the variety of active substances [1,2]. Therefore, they have great potential to become an energy storage device for the next generation of grid-scale applications. Different from the “rocking chair” mechanism of mildly acidic aqueous zinc-ion batteries and the mechanism involving only surface/near surface reactions in supercapacitors, the energy storage mechanism in alkaline aqueous zinc-based batteries mainly originates from conversion reactions [3,4]. The metal cations in the cathode material and the OH^−^ in the electrolyte undergo a redox reaction, and the low-valence transition metals are oxidized to a high-valence state to release electrons during the charging process, and reduced to a low-valence state during the discharge process. Due to the inability to maintain a constant and uniform environment on the electrode surface and the electrolyte during the dissolution and deposition process of zinc in alkaline aqueous solutions, it is inevitable that zinc dendrites will form, accompanied by side reactions, which have adverse effects on the capacity, Coulombic efficiency, and cycle life of the battery [5,6]. Obtaining high-performance alkaline zinc-ion battery cathode materials remains a challenge.

Transition metal phosphides exhibit better electrochemical activity because they have better conductivity than transition metal oxides. Low-temperature phosphating can introduce oxygen vacancies and phosphates into transition metal oxides, effectively improving the electrochemical performance [7]. However, due to the susceptibility of the structure of transition metal phosphating materials to corrosion by alkaline electrolytes, their cyclic stability and rate performance are relatively poor. Metal phosphides are encapsulated in a carbon matrix by using carbon derived from metal–organic frameworks (MOFs), which can improve the electrochemical activity of electrode materials while maintaining their structural framework during charging and discharging, and improve the cycle stability of the battery [8]. Most MOF derivatives have the problem of structural collapse and material agglomeration, which reduces the number of effective active site, blocks ion diffusion channels, and reduces mass transfer kinetics [9]. Direct growth of MOFs on conductive substrates is an effective measure to solve this problem. Using the Co MOF directly grown on foam nickel as a template, we obtained a self-supporting hollow nano-sheet array structure through ion exchange, etching and calcination of NiCo_2_O_4_@Co_3_O_4_. Subsequently, by controlling the amount of NaH_2_PO_2_ used during phosphating treatment, a P-NiCo_2_O_4_@Co_3_O_4_ with rich oxygen vacancy and phosphate solution was obtained. The hollow and porous structure provided a good pathway for electron transfer and electrolyte diffusion. The introduction of oxygen vacancies and phosphates further improved the conductivity of electrons/ions and redox kinetics. Using it as a positive electrode material for alkaline zinc-based batteries has achieved excellent electrochemical performance making it a highly promising candidate material for positive electrodes.

## 2. Results and Discussion

**Figure 1:**
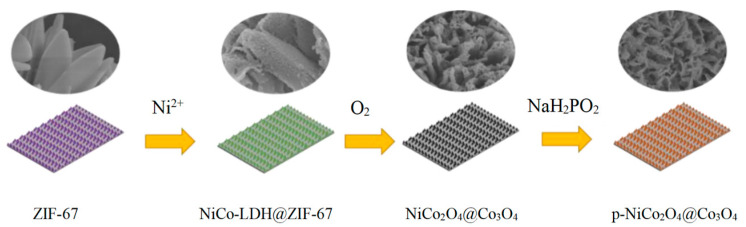
The design strategy of p-NiCo_2_O_4_@Co_3_O_4_.

The design strategy of p-NiCo_2_O_4_@Co_3_O_4_ is shown in Figure 1. First, ZIF-67 was deposited on the surface of foam nickel. Then we used Ni(NO_3_)_2_ solution for ion etching. This process was mainly controlled by the hydrolysis of Ni(NO_3_)_2_. Due to the weak alkalinity of the ligand, ZIF-67 reacted with the H^+^ produced by hydrolysis, releasing Co^2+^. The generated Co^2+^ was partially oxidized by NO_3_^−^ to Co^3+^. Then, Co^2+^/Co^3+^ diffused outward and co precipitated with Ni^2+^ on the ZIF-67 surface to form NiCo-LDH, thereby forming NiCo-LDH@ZIF-67 core shell structure.

Ni^2+^ + 2H_2_O ↔ Ni(OH)_2_ + 2H^+^

**Figure 2:**
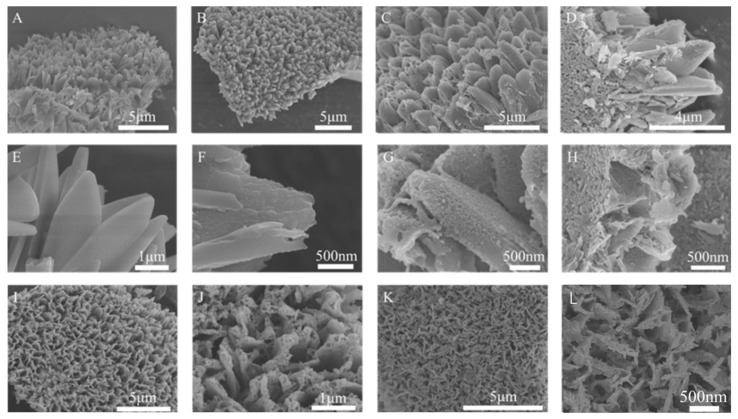
SEM images of (**A**,**E**) ZIF-67; (**B**,**F**) NiCo-LDH@ZIF-67-5; (**C**,**G**) NiCo-LDH@ZIF-67-10; (**D**,**H**) NiCo-LDH@ZIF-67-15; (**I**,**J**) NiCo_2_O_4_@Co_3_O_4_; and (**K**,**L**) P-NiCo_2_O_4_@Co_3_O_4_-2.

The morphology and composition of the products were characterized by SEM. The prepared ZIF-67 formed an array structure on the surface of foam nickel (Figure 2A). The magnified image in Figure 2E exhibits the ZIF-67 has a smooth surface. Figure 2B,F show the morphologies of the sample after 5 min of Ni(NO_3_)_2_ etching, which indicate that the array structure and sheet morphology of ZIF-67 were maintained, and the surface became rough, indicating the growth of new substances. The etching time plays a crucial role in controlling the morphology of ZIF-67. Extending the etching time to 10 min would significantly increase the porosity (Figure 2C,G). A long etching time (15 min) leads to the excessive growth of nanosheets and the destruction of the two-dimensional array. From the broken array part, it can be seen that the material has a hollow structure (Figure 2D,H). After being annealed in air, the array morphology remains intact, as shown in Figure 2I,J, and the gaps between the arrays have increased. A clear hollow feature can be seen from the pores at the top of the array, indicating the formation of hollow and porous NiCo_2_O_4_@Co_3_O_4_-2 array structures. The SEM images of the phosphated P-NiCo_2_O_4_@Co_3_O_4_-2 are shown in Figure 2K,L; it has retained the original array structure of NiCo_2_O_4_@Co_3_O_4_, and the surface has become smoother which indicates a reduction in porosity.

**Figure 3:**
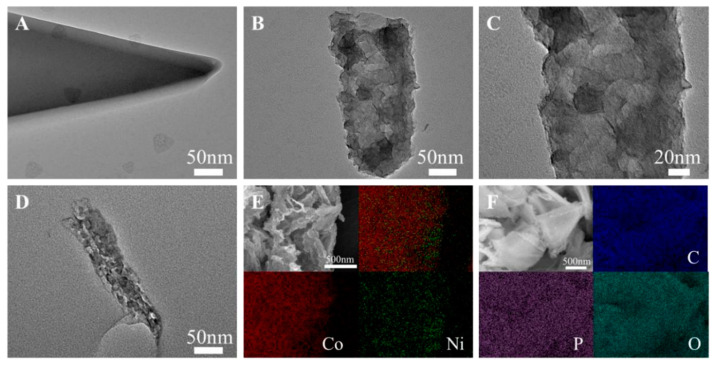
TEM images of the ZIF-67 (**A**); NiCo-LDH@CoMOF-10 ((**B**,**C**) is the enlarged image); P-NiCo_2_O_4_@Co_3_O_4_-2 (**D**); SEM element mapping images of NiCo_2_O_4_@Co_3_O_4_ (**E**); and P-NiCo_2_O_4_@Co_3_O_4_-2 (**F**).

Transmission electron microscopy (TEM) further reveals the evolution process from solid ZIF-67 nanosheets to hollow nanosheets. As shown in Figure 3A, ZIF-67 exhibits a nanosheet morphology, with a smooth surface and uniform internal structure. After being etched in Ni(NO_3_)_2_ solution, the morphology is significant changed (Figure 3B), retaining the original skeleton structure and presenting an uneven layered structure, with the internal hollow structure clearly visible. As shown in Figure 3C, the enlarged image shows that the uneven internal structure indicates an uneven etching process, which is related to the microstructure orientation of ZIF-67. Figure 3D shows the P-NiCo_2_O_4_@Co_3_O_4_-2 obtained after phosphating and calcination, which preserves the hollow and porous structure of NiCo-LDH@CoMOF-10 well. An optimized etching structure results in a more uniform distribution of elements. The formation of a hollow and porous structure is more conducive to electrolyte impregnation and transport. The element distribution image of NiCo_2_O_4_@Co_3_O_4_ is shown in Figure 3E. From the distribution of all elements in the upper right corner, it can be seen that Ni element is distributed throughout the entire sample, and its surface boundary exceeds that of Co element. This indicates that Ni ion gradually penetrates into the interior of ZIF-67 and forms a new substance, NiCo-LDH, on the surface during the etching process, which is consistent with our design strategy.

**Figure 4:**
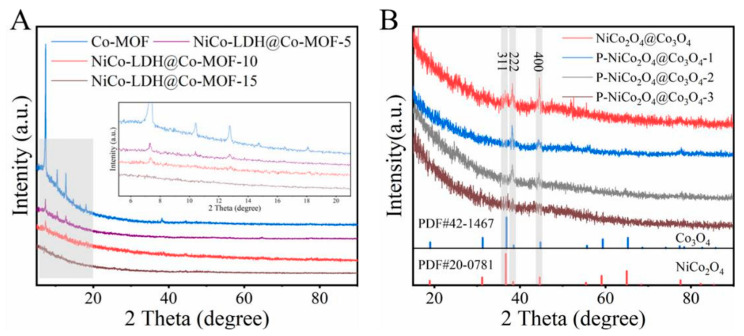
(**A**) The XRD patterns of ZIF-67, NiCo-LDH@ZIF-67-5, NiCo-LDH@ZIF-67-10, and NiCoLDH@ZIF-67-15. (**B**) The XRD patterns of NiCo_2_O_4_@Co_3_O_4_, P-NiCo_2_O_4_@Co_3_O_4_-1, P-NiCo_2_O_4_@Co_3_O_4_-2, and P-NiCo_2_O_4_@Co_3_O_4_-3.

The phase composition transformation during the preparation process was further studied using X-ray diffraction (XRD). The diffraction peak of ZIF-67 in Figure 4A corresponds to the characteristic peak of ZIF-67 reported in the literature [10], confirming the successful synthesis of precursor templates. The ZIF-67 characteristic peaks are weak for samples etched for 5 and 10 min, but completely disappeared after being etched for 15 min, confirming the transformation of ZIF-67 into amorphous NiCo-LDH [11]. The XRD patterns of NiCo_2_O_4_@Co_3_O_4_, P-NiCo_2_O_4_@Co_3_O_4_-1, P-NiCo_2_O_4_@Co_3_O_4_-2, and P-NiCo_2_O_4_@Co_3_O_4_-3 in Figure 4B indicate that the diffraction peaks of NiCo_2_O_4_@Co_3_O_4_ are denoted to Co_3_O_4_ (PDF # 42-1467) and NiCo_2_O_4_ (PDF # 20-0781), respectively. No diffraction peaks related to NiO or CoO are observed, indicating that the obtained sample is a composite of NiCo_2_O_4_ and Co_3_O_4_. The diffraction peak positions of the P-NiCo_2_O_4_@Co_3_O_4_-2 are the same as NiCo_2_O_4_@Co_3_O_4_, indicating that no phase transition occurred during the phosphating process. However, the peak intensity of the sample after phosphating decreased significantly due to the presence of surface phosphate ions, indicating a decrease in crystallinity of the sample after phosphating [12], and no diffraction peaks related to CoP were found. In addition, it was found that with the increase in the amount of NaH_2_PO_2_, the diffraction peak of the NiCo_2_O_4_@Co_3_O_4_ gradually weakened until it disappeared.

**Figure 5:**
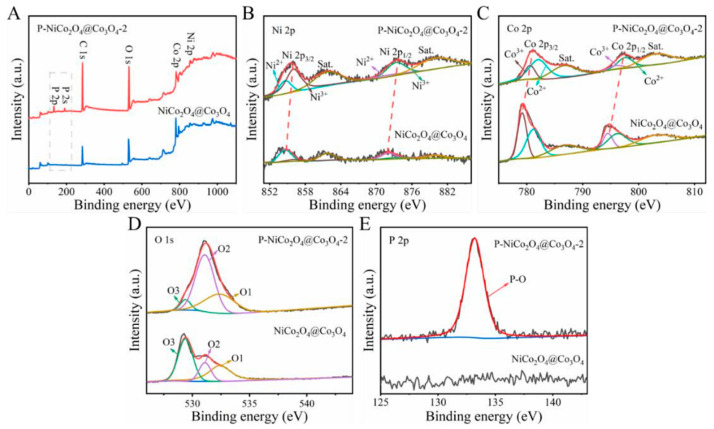
XPS spectrum of P-NiCo_2_O_4_@Co_3_O_4_-2 and NiCo_2_O_4_@Co_3_O_4_: (**A**) survey spectrum; (**B**) Ni 2p; (**C**) Co 1s; (**D**) O 1s; and (**E**) P 2p.

The surface elemental composition and valence states of the NiCo_2_O_4_@Co_3_O_4_ and P-NiCo_2_O_4_@Co_3_O_4_-2 are characterized by XPS. Figure 5A shows the XPS full spectra of two samples; compared with NiCo_2_O_4_@Co_3_O_4_-2, the significant P 2s and P 2p peaks in P-NiCo_2_O_4_@Co_3_O_4_-2 demonstrate the successful introduction of phosphorus element [13,14]. The O1s spectra of the two samples are shown in Figure 5B, and both contain three characteristic peaks: O1 (532.4 eV), O2 (531.1 eV), and O3 (529.4 eV). O1 corresponds to hydroxyl oxygen adsorbed on the surface of the material, O2 corresponds to oxygen defects, and O3 corresponds to metal oxygen bonds [15,16,17]. Compared to NiCo_2_O_4_@Co_3_O_4_, P-NiCo_2_O_4_@Co_3_O_4_-2 exhibits a significant increase in the peak area of O2, indicating an increase in oxygen defects. Oxygen vacancies are effective to improve the conductivity of electrode materials to enhance the reaction kinetics of electrode materials [18,19,20]. At the same time, the peak area of O3 significantly decreases due to the increase in oxygen vacancy concentration and the weakening of metal oxygen bonds after phosphating. Figure 5C shows the high-resolution Co 2p spectrum, with spin orbitals splitting into Co 2p_3/2_ and Co 2p_1/2_. The characteristic peaks at 780.6 and 796.0 eV are attributed to Co^3+^, and the characteristic peaks with a binding energy of 782.1 and 797.6 eV belong to Co^2+^ [21,22]. The peaks located at 786.7 and 803.1 eV are related to two satellite peaks. Compared to NiCo_2_O_4_@Co_3_O_4_, the Co^3+^ (2p) peaks of P-NiCo_2_O_4_@Co_3_O_4_-2 are shifted to the direction of high-binding energy, indicating that some Co^3+^ ions are reduced to Co^2+^ [23] during the phosphating treatment. The Ni2p spectrum is split into Ni^2+^ (872.0 and 854.6 eV) and Ni^3+^ (873.3 and 856 eV), and the peaks at 861.7 and 879.9 eV belong to the satellite peak of Ni (Figure 5D). In addition, comparing P-NiCo_2_O_4_@Co_3_O_4_-2 with NiCo_2_O_4_@Co_3_O_4_-2, the Ni 2p peaks of Ni^2+^ are shifted to the direction of high-binding energy, indicating that some Ni^2+^ ions are oxidized to Ni^3+^ after the phosphating treatment.

**Figure 6:**
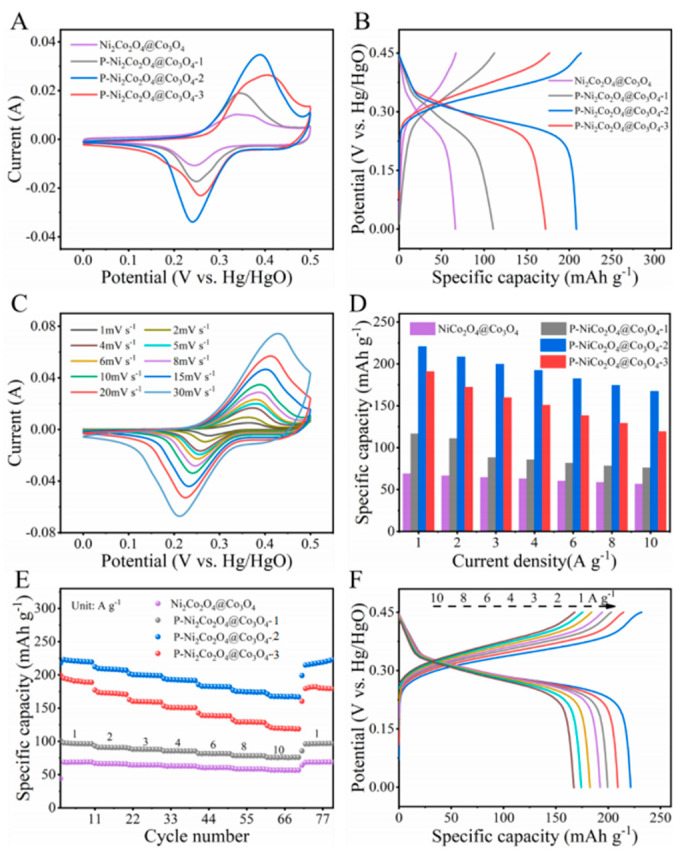
(**A**) CV curves at 10 mV s^−1^; (**B**) GCD curves at 2 A g^−1^; (**D**,**E**) rate performance of NiCo_2_O_4_@Co_3_O_4_, P-NiCo_2_O_4_@Co_3_O_4_-1, P-NiCo_2_O_4_@Co_3_O_4_-2, and P-NiCo_2_O_4_@Co_3_O_4_-3 with current density from 1 to 10 A g^−1^; (**C**) CV curves of P-NiCo_2_O_4_@Co_3_O_4_-2 at various scan rates; and (**F**) GCD curves of P-NiCo_2_O_4_@Co_3_O_4_-2 with current density from 1 to 10 A g^−1^.

The CV curves of NiCo_2_O_4_@Co_3_O_4_, P-NiCo_2_O_4_@Co_3_O_4_-1, P-NiCo_2_O_4_@Co_3_O_4_-2, and P-NiCo_2_O_4_@Co_3_O_4_-3 in a three electrode system in 5 M KOH solution at a scanning rate of 10 mV s^−1^ are shown in Figure 6A, and all samples exhibit a pair of redox peaks, which can be attributed to the reversible Faraday conversion process of MO and MOOH (M=Co, Ni) in an alkaline electrolyte [24]. The GCD curves (Figure 6B) of four samples at a current density of 2 A g^−1^ all exhibit a charge–discharge plateau at the corresponding potential windows, which is consistent with the CV curve [25]. The peak current and integral area of the CV curves of the three phosphating treated samples are greater than that of the untreated sample NiCo_2_O_4_@Co_3_O_4_, which is consistent with the capacity relationship of the GCD curves, indicating that phosphating treatment greatly improves the electrochemical activity of the electrode material [26]. Among them, the integrated area of P-NiCo_2_O_4_@Co_3_O_4_-2 is the largest, and the maximum specific capacity reaches 209 mAh g^−1^. The CV curve shapes (Figure 6C) of P-NiCo_2_O_4_@Co_3_O_4_-2 at different scan rates (1–30 mV s^−1^) remain almost unchanged as the scan rate increases, indicating that the material possesses excellent rate performance and reversibility.

The discharge specific capacities of the four samples at current densities of 1, 2, 3, 4, 6, 8, and 10 A g^−1^ are shown in Figure 6D, where those of NiCo_2_O_4_@Co_3_O_4_ are the lowest, that is 68.8, 66.3, 64.4, 62.9, 60.2, 58.5, and 56.5 mAh g^−1^, respectively. The discharge specific capacities of P-NiCo_2_O_4_@Co_3_O_4_-1, P-NiCo_2_O_4_@Co_3_O_4_-3, and P-NiCo_2_O_4_ @Co_3_O_4_-2 are 116.5, 110.7, 88.1, 85.5, 81.5, 78.3, and 75.9 mAh g^−1^; 190.8, 172.2, 159.6, 150.7, 138.3, 129.1; and 119.1 mAh g^−1^; 220.6, 208.4, 199.6, 192.3, 182.4, 174.5, and 167.3 mAh g^−1^, respectively. When the current density returns to 1 A g^−1^, the discharge specific capacity of all samples can return to its original value (Figure 6E), indicating that they possess excellent rate performance. Among them, P-NiCo_2_O_4_@Co_3_O_4_-2 is the best, and can work reversibly with satisfied specific capacity at the high current density.

**Figure 7:**
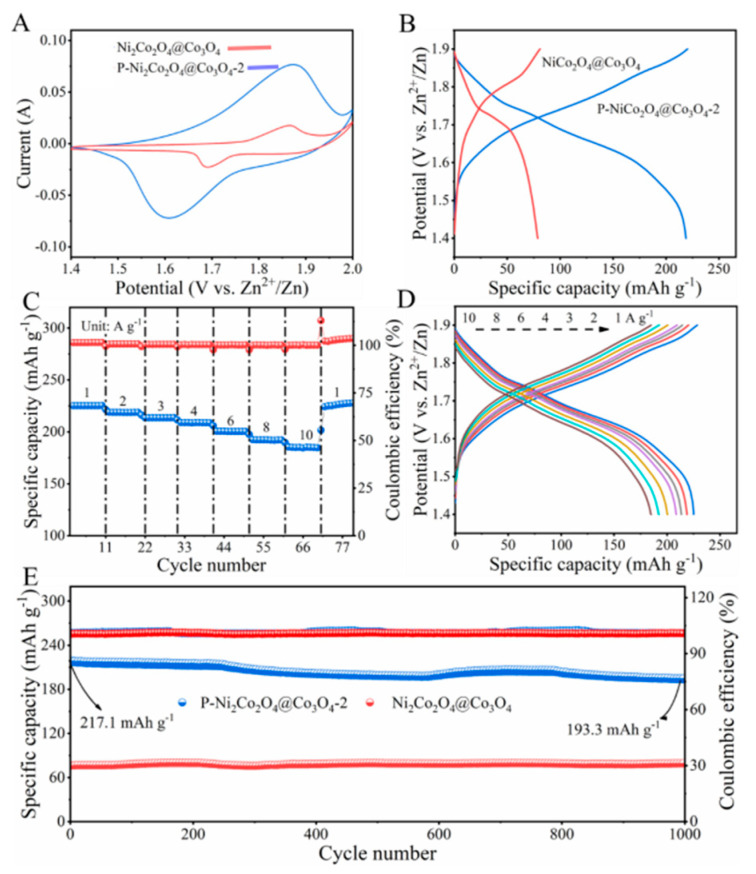
(**A**) CV curves at 15 mV s^−1^; (**B**) GCD curves at 3 A g^−1^ of P-NiCo_2_O_4_@Co_3_O_4_-2//Zn and NiCo_2_O_4_@Co_3_O_4_//Zn; (**C**) rate performance; (**D**) GCD curves of P-NiCo_2_O_4_@Co_3_O_4_-2//Zn with current density from 1 to 10 A g^−1^; and (**E**) Cycling performance of P-NiCo_2_O_4_@Co_3_O_4_-2//Zn and NiCo_2_O_4_@Co_3_O_4_//Zn at 3A g^−1^, the interwoven blue and red lines in the upper part of the figure is the Coulomb efficiency curve.

Using a zinc plate as the anode and a mixture solution of 5 M KOH and 0.3 M Zn(Ac)_2_ as the electrolyte, the P-NiCo_2_O_4_@Co_3_O_4_-2//Zn battery is assembled. Simultaneously, the NiCo_2_O_4_@Co_3_O_4_//Zn battery is set as a comparison. Figure 7A shows the CV curves of P-NiCo_2_O_4_@Co_3_O_4_-2//Zn and NiCo_2_O_4_@Co_3_O_4_//Zn at a scan rate of 15 mV s^−1^. The redox peak can be attributed to the reversible conversion of Ni^3+^/Ni^2+^, Co^3+^/Co^2+^, and even Co^3+^/Co^4+^ in alkaline electrolytes [27]. The reaction mechanism can be described by the following equation [28]:

Co_3_O_4_ + OH^−^ + H_2_O ↔ 3 CoOOH + 3 e^−^ (1)

NiCo_2_O_4_ + OH^−^ + H_2_O ↔ NiOOH + 2 CoOOH + 3 e^−^ (2)

CoOOH + OH^−^ ↔ CoO_2_ + H_2_O + e^−^ (3)

2 NiCo_2_O_4_ + 3 Zn(OH)_4_^2−^ ↔ 2 NiOOH +4 CoO_2_ + 3 Zn + 6 OH^−^ + 2 H_2_O (4)

Co_3_O_4_ + 2 Zn(OH)_4_^2−^ ↔ 3 CoO_2_ + 2 Zn + 4 OH^−^ + 2 H_2_O (5)

During the charging process, Zn(OH)_4_^2−^ is reduced to Zn, releasing OH^−^ into the electrolyte. At the same time, NiCo_2_O_4_ and Co_3_O_4_ react with OH^−^ in the electrolyte and convert into NiOOH and CoO_2_. During the discharge process, Zn reacts with OH^−^ in the electrolyte to convert into Zn(OH)_4_^2−^. At the same time, NiOOH and CoO_2_ are reduced to corresponding low-valent oxides, releasing OH^−^ [29,30].

Figure 7B shows the GCD curves of P-NiCo_2_O_4_@Co_3_O_4_-2//Zn and NiCo_2_O_4_@Co_3_O_4_//Zn at 3 A g^−1^ current density. The discharge platform of the P-NiCo_2_O_4_@Co_3_O_4_-2//Zn batteries is significantly longer than that of the NiCo_2_O_4_@Co_3_O_4_//Zn batteries, exhibiting a higher discharge specific capacity of 218 mAh g^−1^. The P-NiCo_2_O_4_@Co_3_O_4_-2//Zn batteries exhibit excellent rate performance (Figure 7C). The discharge specific capacity of the battery is 225.3, 218.8, 213.5, 208.9, 200.6, 192.3, and 184.6 mAh g^−1^ at current densities 1, 2, 3, 4, 6, 8, and 10 A g^−1^, respectively. When the current density drops from 10 to 1 A g^−1^, the discharge specific capacity of the P-NiCo_2_O_4_@Co_3_O_4_-2//Zn batteries reaches back to 226.5 mAh g^−1^, indicating that P-NiCo_2_O_4_@Co_3_O_4_-2 possesses particularly excellent reversibility. Figure 7D shows the GCD curves of the P-NiCo_2_O_4_@Co_3_O_4_-2//Zn batteries at the corresponding current density. Each charging and discharging curve has a voltage platform, and the position of the platform roughly matches the position of the redox peak in the CV curve. When the current density changes from 1 to 10 A g^−1^, the corresponding voltage plateau does not change much, proving that P-NiCo_2_O_4_@Co_3_O_4_-2//Zn batteries have excellent structural stability. Figure 7E shows the cyclic stability of two batteries at 3 A g^−1^. The initial capacity of P-NiCo_2_O_4_@Co_3_O_4_-2//Zn is 217.1 mAh g^−1^, and after 1000 cycles of charging and discharging, the capacity is 193.3 mAh g^−1^, with a retention rate of 89% and good stability. This is due to the direct growth of MOF precursor on foam nickel substrate. During etching, phosphating, and calcination, MOF-derived carbon materials have fixed P-NiCo_2_O_4_@Co_3_O_4_-2. Therefore, the structure of the electrode material is maintained during the charging and discharging process, avoiding structural collapse.

**Figure 8:**
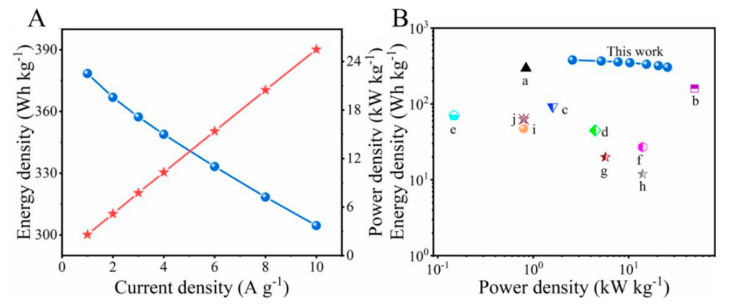
(**A**) Power density(red star) and energy density(blue ball) of P-NiCo_2_O_4_@Co_3_O_4_-2//Zn at different current densities and (**B**) Ragone plot of P-NiCo_2_O_4_@Co_3_O_4_-2//Zn, the details of a, b, c, d, e, f, g, h, i and j are given in Table 1, respectively.

Figure 8A describes the relationship between power density and energy density of P-NiCo_2_O_4_@Co_3_O_4_-2//Zn batteries at different current densities, with a maximum energy density of 378.4 Wh kg^−1^ and a corresponding power density of 2.6 kW kg^−1^. Even at the highest power density of 25.5kW kg^−1^, its energy density still reaches up to 304.5 Wh kg^−1^. Figure 8B shows the Ragon diagram of battery energy density and power density. The energy density of P-NiCo_2_O_4_@Co_3_O_4_-2//Zn batteries is superior to many reported aqueous zinc-based batteries, and the power density is also significantly better than some supercapacitors (Table 1).

**Figure 9:**
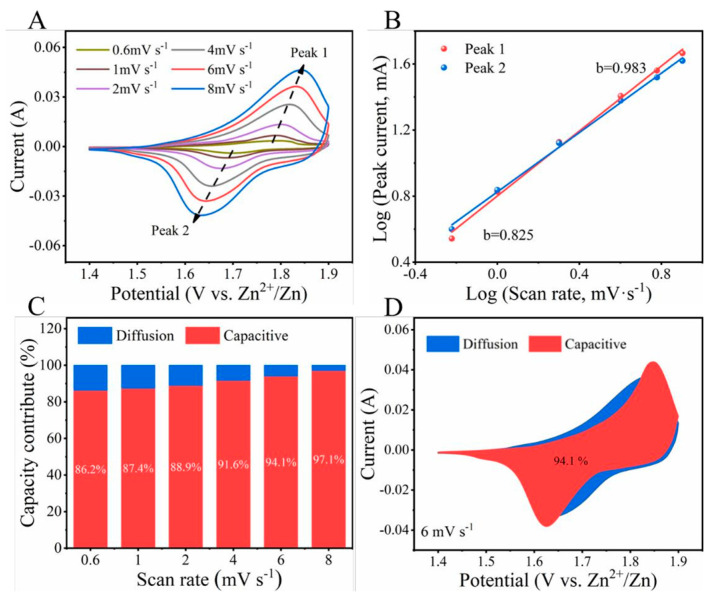
(**A**) CV curves of P-NiCo_2_O_4_@Co_3_O_4_-2//Zn at various scan rates; (**B**) relationship between log *i* and log *v* at specific peak currents; (**C**) the percentage of capacity contribution (red region) at different scan rates; and (**D**) CV curve P-NiCo_2_O_4_@Co_3_O_4_-2//Zn electrode with the capacitive contribution (red region) at 6 mV s^−1^.

In order to investigate the electrochemical kinetics behavior of the P-NiCo_2_O_4_@Co_3_O_4_-2//Zn battery, CV curves were obtained at 0.6, 1, 2, 4, 6, and 8 mV s^−1^ within the potential window between 1.4 and 1.9 V. Figure 9A exhibits that the redox peaks shift but the CV curves did not show significant deformation, remaining highly reversible with the increase in scan rates. Normally, the Dunn power law relationship, *i* = a*v*^b^, is used to analyze the surface capacitive and diffusion-controlled processes of a battery, where *i* is the peak current, *v* is the scan rate, and a and b are adjustable parameters. The b value of the oxidation peak of the P-NiCo_2_O_4_@Co_3_O_4_-2//Zn battery is 0.983, and the reduction peak is 0.825, indicating that the charge storage process belongs to a mixed control process, which includes both diffusion control behavior and capacitance control behavior. But the value of b is closer to one, indicating that capacitor control dominates the charge storage process.

In order to further determine the contribution of these two different mechanisms to capacity, the percentages of capacitance contribution and diffusion contribution at different scanning rates were calculated (Figure 9C). It can be seen that the proportion of capacitance contribution increases with the increase of scan rate, possibly due to slow charge transfer during the diffusion process and the inability to respond quickly to potential changes at high scan rates. Therefore, the current contributed by this part will sharply decrease as the scanning rate increases. It is worth noting that pseudocapacitance has a large proportion at different scan rates, reaching 94.1% at a scan rate of 6 mV s^−1^ (Figure 9D), indicating that P-NiCo_2_O_4_@Co_3_O_4_-2//Zn batteries exhibit fast charge transfer kinetics, which is also why they exhibit high rate performance.

**Figure 10:**
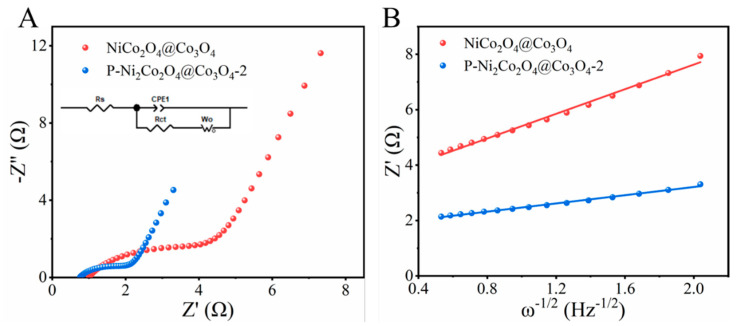
(**A**) Nyquist plots of NiCo_2_O_4_@Co_3_O_4_//Zn and P-NiCo_2_O_4_@Co_3_O_4_-2//Zn (inset is the electrical equivalent circuit) and (**B**) the linear relationships between *Z*′ and ω^−1/2^ of the NiCo_2_O_4_@Co_3_O_4_//Zn and P-NiCo_2_O_4_@Co_3_O_4_-2//Zn in the low-frequency region.

The EIS curve and the fitted equivalent circuit model are shown in Figure 10A. The high-frequency region is typical of a semicircle, while the low-frequency region is a diagonal line. From the fitting results, the semicircle diameter of P-NiCo_2_O_4_@Co_3_O_4_-2 in the high-frequency region is significantly smaller than that of NiCo_2_O_4_@Co_3_O_4_, indicating that P-NiCo_2_O_4_@Co_3_O_4_-2 has a smaller charge transfer resistance; thus the conductivity of P-NiCo_2_O_4_@Co_3_O_4_-2 is significantly increased. This is because the introduction of oxygen vacancies and phosphate ions after phosphating increases conductivity and improves the electrochemical reaction kinetics of the electrode material. In addition, the ion diffusion rate is also an important factor affecting electrode performance. Generally, the slope σ of the relationship curve between the impedance real part *Z*’ and ω^−1/2^ is used to evaluate the ion diffusion rate; the smaller the size, the greater the ion diffusion rate. The linear fitting results of the values are shown in Figure 10B. The σ of P-NiCo_2_O_4_@Co_3_O_4_-2 is smaller than that of NiCo_2_O_4_@Co_3_O_4_, i.e., P-NiCo_2_O_4_@Co_3_O_4_-2 has a higher ion diffusion coefficient. According to the Randles–Sevcik Equation and the CV curve, it can be further obtained that the diffusion coefficients of P-NiCo_2_O_4_@Co_3_O_4_-2 and NiCo_2_O_4_@Co_3_O_4_ are 6.35 × 10^−14^ and 1.65 × 10^−14^, respectively.

## 3. Materials and Methods

### 3.1. Preparation of ZIF-67

2-Methylimidazole solution (20 mL, 0.40 M) was quickly added to Co (NO_3_)_2_ solution (20 mL, 0.05 M). Then, clean foam nickel (NF) was vertically put into the mixed solution, stood at room temperature for 4 h, taken out, clean with deionized water, and vacuumed dry at 60 °C for 24 h to obtain ZIF-67@NF.

### 3.2. Preparation of NiCo-LDH@ZIF-67

ZIF-67@NF was added to Ni(NO_3_)_2_ solution (50 mL, 0.01 M), etched for 10 min, then washed thoroughly, and dried at 60℃ for 24 h. This was recorded as NiCo-LDH@ZIF-67-10. At the same time, the etching time was changed to 5 min and 15 min, respectively, and the corresponding etching products were denoted as NiCo-LDH@ZIF-67-5, NiCo-LDH@ZIF-67-15.

### 3.3. Preparation of P-NiCo_2_O_4_@Co_3_O_4_

The obtained NiCo-LDH@ZIF-67-10 was placed in a tubular furnace, heated up to 300 °C for 2 h; the heating rate is 1 °C min^−1^ to obtain NiCo_2_O_4_@Co_3_O_4_. The obtained NiCo_2_O_4_@Co_3_O_4_ and 0.01 g of NaH_2_PO_2_ were placed in the upstream and downstream positions of the porcelain boat, respectively, in a tubular furnace, and kept at 300 °C for 2 h in a N_2_ atmosphere to obtain P-NiCo_2_O_4_@Co_3_O_4_-2. The loading capacity is approximately 1.2 mg. The dosage of NaH_2_PO_2_ was changed to 0.005 and 0.02 g to obtain P-NiCo_2_O_4_@Co_3_O_4_-1 and P-NiCo_2_O_4_@Co_3_O_4_-3, respectively.

## 4. Conclusions

In this paper, the cathode material P-NiCo_2_O_4_@Co_3_O_4_ with excellent alkaline aqueous zinc-ion battery performance is obtained from the direct growth on the foam nickel in combination with ion-etching technology. This method improves the structural stability of P-NiCo_2_O_4_@Co_3_O_4_, creates more ion transmission channels, and highly enhances the ion diffusion rate and conductivity. The capacitance contribution dominates the charging and discharging process, which greatly improves the power density of the battery while maintaining an excellent energy density. It obtains an excellent energy density of 304.5 Wh kg^−1^ at a super-high power density of 25.5 kW kg^−1^, exhibiting excellent application potential.

## Figures and Tables

**Table 1:** Comparison of energy density and power density of P-NiCo_2_O_4_@Co_3_O_4_-2//Zn battery with other materials in the literature.

Materials	Energy Density (Wh kg^−1^)	Power Density (kW kg^−1^)	Literature
a, R-Co_3_O_4_//Zn	295.5	0.84	[31]
b, NiCo_2_O_4_//Zn	159.4	49	[32]
c, VS_2_//Zn	92	1.6	[33]
d, NaV_2_(PO_4_)_2_F_3_//Zn	44.7	4.47	[34]
e, MoS_2_//Zn	148.2	70.5	[35]
f, Co_0.1_Ni_0.9_P//AC	14	27	[36]
g, Ni-CoP/POx//RGO	5.7	19.9	[37]
h, NiMoP@CoCH//a-MEGO	14	11.9	[13]
i, NiCo_2_S_4_//AC	0.7935	47.29	[14]
j, ZnCo_2_O_4_//AC	0.7955	63	[38]
This work	304.5	25.5	This work

## Data Availability

Data is contained within the article.
